# The lexical processing of Japanese collocations by Chinese Japanese-as-a-Foreign-Language learners: An experimental study by manipulating the presentation modality, semantic transparency, and translational congruency

**DOI:** 10.3389/fpsyg.2023.1142411

**Published:** 2023-04-04

**Authors:** Qichao Song, Xiaodong Fei, Norio Matsumi

**Affiliations:** ^1^Graduate School of Humanities and Social Sciences, Hiroshima University, Hiroshima, Japan; ^2^Beijing Center for Japanese Studies, Beijing Foreign Studies University, Beijing, China

**Keywords:** Japanese collocational processing, translational congruency, Chinese JFL learners, presentation modality, semantic transparency

## Abstract

**Introduction:**

Research on collocations has become an essential issue in L2 acquisition and cognitive psychology. Previous studies have mainly focused on phonographic languages such as English, Swedish, and German, and primarily discussed the effect of semantic transparency and translational congruency. However, these studies have lacked (1) an analysis of the interactions between presentation modalities (visual vs. auditory) and the semantic transparency and translational congruency, and (2) a discussion of an ideographic language, such as Chinese and Japanese.

**Methods:**

We conducted an experiment with 36 Chinese Japanese-as-a-Foreign-Language learners to examine the processing of Japanese collocations. In the experiment, we manipulated the presentation modality, semantic transparency, and translational congruency during a lexical judgment task.

**Results:**

Data analysis using linear mixed-effects models revealed the following. (1) In both conditions of semantic transparency and translational congruency, the auditory presentation was associated with longer reaction times than the visual presentation. (2) In the visual presentation condition, neither semantic transparency nor translational congruency showed significant effects. (3) In the auditory presentation condition, the reaction time for collocations with high semantic transparency tended to be longer than that for collocations with medium semantic transparency and significantly longer than that for collocations with low semantic transparency. The reaction time for collocations with congruent translation was longer than that for collocations with incongruent translation.

**Discussion:**

These results support the dual-route model of Japanese collocational processing by Chinese Japanese-as-a-Foreign-Language learners. Our findings suggest that whether the analytic or holistic processing dominates is closely related to the learners’ knowledge of Chinese and Japanese *Kanji* words and strongly influenced by the presentation modality, semantic transparency, and translational congruency.

## 1. Introduction

When learners engage in language activities in a second language (L2), grammatical knowledge and vocabulary knowledge play an essential role (Bernhardt, 2005; Jeon and Yamashita, 2014). Collocation is one of the key points in assessing learners’ vocabulary proficiency (e.g., Pawley and Syder, 1983; Nation, 2013; Koizumi and In’nami, 2020; Du et al., 2022). However, it has been noted that mastering collocations is challenging, even for advanced foreign language learners (Laufer and Waldman, 2011). Therefore, research on collocations has attracted attention as an essential issue in L2 acquisition and cognitive psychology (Kjellmer, 1991).

Previous studies have shown that factors such as frequency of use, native language (L1), and L2 proficiency affect the processing of L2 collocations (e.g., Sprenger et al., 2006; Yamashita and Jiang, 2010; Wolter and Gyllstad, 2011; Wolter and Yamashita, 2015, 2018; Zhang and Fang, 2020; Fei and Song, 2021; Jiang, 2022; Song and Fei, 2022). In particular, the research focuses on the co-occurrence strength of each constituent word of the collocation and the bilingual translational relationship, i.e., semantic transparency and translational congruency. Semantic transparency is the degree to which the meaning of a collocation can be inferred from its parts, while translational congruency refers to the fact that the collocation meaning can be translated or inferred with the aid of L1 (e.g., Günther et al., 2020; Song and Fei, 2022).

Previous studies have focused on phonographic languages such as English, Swedish and German (e.g., Wolter and Gyllstad, 2011; Garibyan et al., 2022), some involving Japanese learners of English (e.g., Yamashita and Jiang, 2010), and Chinese learners of English (e.g., Zhang and Fang, 2020; Jiang, 2022). However, there is almost no research on Chinese learners of Japanese (Fei and Song, 2021). Since Chinese and Japanese use Chinese characters, an ideographic writing system, it is clear that Chinese learners of Japanese are strongly influenced by their knowledge of Chinese characters in their lexical processing of Japanese (e.g., Matsumi et al., 2012; Hsieh et al., 2017, 2021; Fei et al., 2022). Therefore, the processing of Japanese collocations consisting of several words by Chinese learners is expected to be complex, and it is possible that different mechanisms would be observed during the processing of Japanese collocations in Chinese learners.

Based on the research results of L2 collocational processing, especially the research results of the collocational processing by Chinese English learners and the lexical processing by Chinese Japanese-as-a-Foreign-Language (JFL) learners, this study aims to clarify the effects of semantic transparency, an essential linguistic characteristic, and translational congruency, which is closely related to bilingualism, upon both visual and auditory presentation of test items. In this study, we explore the lexical processing of Japanese collocations, provide new empirical evidence for the study of collocational processing from the perspective of ideographic characters, and offer suggestions for teachers on how to improve Chinese JFL learners’ acquisition of Japanese collocations.

## 2. Literature review

### 2.1. Definition and classification of collocations

From a broad perspective, a collocation is defined as the co-occurrence relationship between words (Sinclair, 1991). In other words, collocations include lexical, grammatical, and contextual elements and are characterized by structural stability, formal unity, and usage restrictiveness (Wray, 2002). For example, the Japanese verb “投げる (nageru), throw” is used in collocations such as “石を投げる (ishi-wo-nageru), throw a stone”; “視線を投げる (shisen-wo-nageru), throw a glance”; “筆を投げる (fude-wo-nageru), throw the pen and give up writing”; “匙を投げる (saji-wo-nageru), beyond remedy.” All these collocations exist in Japanese but differ in co-occurrence relationship and the relationship between individual constituents and the overall meaning.

According to the definitions in previous studies of phonographic languages (Nattinger and DeCarrico, 1992), collocations in Japanese were classified into three types by Miyoshi (2007). The three types are collocations with high semantic transparency, collocations with medium semantic transparency, and collocations with low semantic transparency. Collocations with high semantic transparency (“石を投げる”) are free word combinations characterized by the lowest strength of linkage between the individual constituent words. Collocations with medium semantic transparency (“視線を投げる”) are somewhat fixed word combinations, and the strength of linkage between its individual constituents is medium. Collocations with low semantic transparency (“筆を投げる”; “匙を投げる”) are wholly fixed word combinations, and the strength of linkage between the individual constituent words is the strongest. Furthermore, collocations with low semantic transparency were subdivided into figurative idioms and genuine idioms. A figurative idiom’s meaning can be inferred from the meanings of its individual constituents (“筆を投げる”), while a genuine idiom’s meaning cannot be inferred (“匙を投げる”).

The above classification is based on the linguistic characteristics of Japanese. How can the above collocations be classified taking into account the relationship between Chinese and Japanese languages? Matsumi et al. (2012) revealed that the morphological, phonological, and semantic information of L1 Chinese characters significantly impacts Chinese learners’ processing of Japanese *Kanji* words. Through behavioral experiments and fMRI scanning, Hsieh et al. (2021) also confirmed that Chinese-Japanese cognate word processing showed longer reaction time and greater activation in the supplementary motor area than L2 control word processing. Therefore, it is essential to classify collocations according to whether or not the Japanese meaning can be inferred from the Chinese meanings of the individual constituent words. Fei and Song (2021) categorized collocations in Japanese into those with matching and non-matching bilingual translations between Chinese and Japanese. In the case of the three types of collocations mentioned above, namely, collocations with high semantic transparency, collocations with medium semantic transparency, and figurative idioms (one subtype of the collocation with low semantic transparency), Chinese learners can infer the overall meaning of the collocation with the aid of L1. In contrast, as for genuine idioms (another subtype of the collocation with low semantic transparency), Chinese learners cannot infer the overall meaning of the collocation with the aid of L1.

More specifically, the semantic transparency of the three collocations “石を投げる” “視線を投げる” “筆を投げる” progressively decreases. “石を投げる” can be translated as “扔掉石头 (rengdiao-shitou),” and “視線を投げる” can be translated as “投去视线 (touqu-shixian),” both retaining the complete or partial original meanings of Chinese characters. Therefore, the overall meaning can be inferred based on the knowledge of L1 Chinese. The literal meaning of “筆を投げる” can be interpreted as “弃笔 (qibi), throw away the pen” and it can be further speculated that its overall meaning is “中途弃写 (zhongtu-qixie).” In contrast, the literal meaning of “匙を投げる,” which is also of low semantic transparency, can be interpreted as “弃勺 (qishao),” and after further speculation can be interpreted as “不吃饭 (bu-chifan), do not eat.” It is impossible to use the knowledge of L1 to guess right about the meaning of this collocation. Thus it belongs to incongruent translational collocation.

According to the above analysis, it is clear that semantic transparency is a linguistic characteristic of the language itself, while translational congruency is a linguistic characteristic involving two languages. On the basis of the existing research results about collocational processing and the specific characteristics of Japanese collocations, this study comprehensively investigates Japanese collocational processing and compares the results with those about the collocational processing of phonographic languages (such as English and German, but not Hindi and Urdu) and the lexical processing of ideographic languages.

### 2.2. Collocational processing

#### 2.2.1. Hypotheses about the collocational processing model

Regarding language processing, Sinclair (1991) proposed two principles: the “open choice principle,” under which constituent words are processed according to grammatical rules, and the “idiom principle,” under which pre-existing linguistic expressions are processed as a single entity. In addition, Wray (2002) identified two patterns of language processing: analytical processing based on syntactic knowledge and holistic processing using formulaic sequences. It is assumed that these two strategies are used properly when processing languages, and the “idiom principle” is said to have the advantage of reducing cognitive burden. Pawley and Syder (1983) argued that since native speakers have more than thousands of formulaic sequences, including the collocations stored in their mental lexicon, they can process language quickly and accurately. In contrast, L2 learners’ mental lexicon stores fewer formulaic sequences and is more inclined to follow the “open choice principle” during L2 processing (Jiang, 2022).

Farrokh (2012) pointed out that in L2 acquisition, learners learn collocations analytically or holistically, depending on the level of semantic transparency. This argument was consistent with the two patterns of language processing mentioned above. In previous studies, there has been debate over whether or not collocations are stored as a whole in the learner’s mental lexicon. The main arguments are the full-listing model (e.g., Seidenberg and Gonnerman, 2000; Jiang and Nekrasova, 2007; Conklin and Schmitt, 2008), the decompositional model (e.g., Schmitt and Underwood, 2004; Brooks and Cid de Garcia, 2015), and the dual-route model (e.g., Sprenger et al., 2006; MacGregor and Shtyrov, 2013; Chen et al., 2020).

Previous studies have discussed collocational processing, mainly using reaction times and accuracy rates as indices. Suppose that the reaction time for formulaic sequences is shorter than that for atypical expressions. In that case, the formulaic sequences are stored in the learner’s mental lexicon as a whole, and then a full-listing model is supported. On the other hand, if there is no significant difference in reaction time for formulaic sequences and atypical expressions, formulaic sequences are also processed based on syntactic knowledge, and the decompositional model is supported. According to Zhang et al. (2021), many studies supported the full-listing model. However, Xu and Wang (2015) argued that the “shorter reaction time” may not be direct evidence of the full-listing model. They pointed out that the highly frequent co-occurrence seen in collocations may result in a stronger linkage between its constituent words, thus improving the efficiency of processing the collocations for L2 learners and reflecting a shorter reaction time. Nevertheless, this does not entirely imply that collocations with short reaction times are stored in the mental lexicon as a lexical entry. Each constituent word may still be stored separately in the mental lexicon, but the processing speed becomes faster because of the strong linkage. This led to the question of the validity of the full-listing model. Therefore, the dual-route model, in which the decompositional model and the full-listing model coexist, was proposed. Many studies tried to find out which processing model is dominant by manipulating various factors (e.g., Sosa and MacFarlane, 2002; Wolter and Gyllstad, 2011; Fang and Zhang, 2021).

Regarding the L2 lexical processing model, Kroll and Stewart (1994) proposed the revised hierarchical model, which has been widely used. This model clearly proposes that the L2 lexicon is independent and shares conceptual representation with L1. This model is suitable for studying languages with relatively independent glyphs and sounds, such as Chinese and Japanese (Fei et al., 2022). Sharing Chinese character representations may make Japanese collocational processing by Chinese learners more closely related to their L1. Therefore, it is necessary to focus on bilingual representation activation when exploring the Japanese collocational processing model. In addition, previous studies mainly explored the selection of the full-listing model or the decompositional model based on reaction time. The revised hierarchical model is also based on reaction time to show the processing path between two languages. Based on these, the present study aims to systematically explore the model of Chinese JFL’s Japanese collocational processing by describing the representational links between Chinese and Japanese bilingual mental lexicon.

#### 2.2.2. Factors affecting L2 collocational processing

Factors affecting L2 collocational processing have been divided into two main categories. They are internal factors such as frequency of use, semantic transparency, and translational congruency, and external factors other than the collocation, such as L2 proficiency, context, and presentation modality.

It has already been shown that frequency of use strongly influences the acquisition and processing of L2, and there is a common understanding among studies to date. Research results showed that the frequency of collocation use had an effect, using eye movements as a measure of eye tracking (e.g., Siyanova et al., 2011) and reaction times (e.g., Wolter and Gyllstad, 2013). More precisely, collocations used more frequently showed a processing advantage over collocations used less frequently.

It was found that semantic transparency affects L2 collocational processing. Collocations with low semantic transparency were observed to be associated with shorter reaction times than collocations with high semantic transparency (Libben et al., 2003; Brooks and Cid de Garcia, 2015; Chen et al., 2020). Collocations with low semantic transparency, such as formulaic sequences and idioms, were more likely to be processed by the full-listing model. On the other hand, results were also reported where semantic transparency did not significantly affect L2 collocational processing (Frisson et al., 2008). In this regard, Zhang et al. (2021) conducted a priming task under different SOA (stimulus onset asynchrony) conditions (200 ms, 400 ms, and 600 ms), and their results suggested that the effect of semantic transparency was weakened by the processing time of collocations. Chen et al. (2020) also showed that the effect of semantic transparency is influenced by factors such as the frequency of use. Therefore, the influence of semantic transparency should be examined together with other factors.

Additionally, it has been shown that translational congruency, which reflects the relationship between L1 and L2, affects collocational processing. It was observed that the reaction time was shorter when the bilingual translation of collocation matched (i.e., congruent translational collocation) than when it did not (e.g., Yamashita and Jiang, 2010; Wolter and Gyllstad, 2011; Zhang, 2017). On the other hand, similar to semantic transparency, some studies reported that translational congruency had no effect on reaction time (e.g., Fang and Zhang, 2019; Fei and Song, 2021). It was noted that the effect of translation congruency is affected by the linguistic distance (i.e., degree of the actual difference) between the two languages (Wolter and Yamashita, 2015) and the judgment criteria for translational congruency (Zhang and Fang, 2020). Studies focused on phonographic language learners, and few discussed about ideographic Chinese characters. Considering the sharing of some Chinese character representations and the short linguistic distance (see Chai and Bao, 2023), different results may be found in Chinese-Japanese bilingual research.

In addition to internal factors due to the linguistic characteristics of collocation, the influence of external factors also cannot be ignored. Pawley and Syder (2000) pointed out that the more fluent the speaker is, the more pauses are placed between phrases, and there are almost no breaks in sound due to pauses or hesitation within phrases. It can be inferred that learners’ L2 proficiency affects their processing of collocations. In this regard, experimental studies that manipulated learners’ L2 proficiency demonstrated that proficiency affects collocational processing and how frequency of use, semantic transparency, and translational congruency function (Wolter and Yamashita, 2018; Zhang and Fang, 2020). Therefore, if language ability is not taken as a factor when exploring collocational processing, it is necessary to control the language ability of participants. Furthermore, it was reported that context affects the influences of semantic transparency and translational congruency on collocational processing (Jiang and Nekrasova, 2007; Cervera and Rosell, 2015). It was shown that context relatively weakly affected semantic transparency’s influence, while it significantly affected translational congruency’s influence on collocational processing (Song and Fei, 2022).

Whether discussing internal or external factors, most of the studies examined the processing of collocations using the visual presentation condition. A previous study using an auditory presentation condition reported that semantic transparency had a strong effect on the processing of Japanese collocations by Chinese learners of Japanese, while translational congruency had a weak effect (Fei and Song, 2021). Zhang and Fang (2020) pointed out that the influence of each factor depends on the experimental paradigm. Further studies are needed to examine how different presentation modalities change the effects of semantic transparency and translational congruency on collocational processing.

### 2.3. Objectives and issues of this study

According to the literature review of previous studies, the following three points became clear. First, most studies dealt with phonographic languages, and only a few studies dealt with languages that use ideographic Chinese characters. Second, the effect of the frequency of use of L2 was consistently observed in all the studies. Third, semantic transparency and translational congruency affected collocational processing in the visually presented condition, but how they affect collocational processing depended on the context. However, the results of using the auditory presentation modality and the interactions between presentation modalities and the linguistic characteristics of collocations have not been extensively investigated. We believe that collocational processing can be clarified by examining these issues. In particular, exploring these issues can further demonstrate how the decompositional model and the full-listing model can co-exist.

Based on these results, this study examines the following three research questions.

RQ1. How do presentation modality (visual vs. auditory) and semantic transparency interact to affect the semantic processing of Japanese collocations?
RQ2. How do presentation modality and translational congruency interact to affect the semantic processing of Japanese collocations?
RQ3. What kinds of Japanese collocational processing models do Chinese JFL learners follow?

## 3. Materials and methods

### 3.1. Participants

A total of 36 advanced Chinese JFL learners (female, 22; male, 14) participated in the experiment. The average age of the participants was 24.7 years old, and all were enrolled in graduate school in China. All participants began studying Japanese in their first year of college, with an average of 5.9 years of Japanese language study. All participants had normal vision (corrected) and hearing. And all had attained Japanese-Language Proficiency Test (JLPT) N1 certificate (the most difficult level, the ability to understand Japanese used in various circumstances). The participants were randomly divided into an auditory presentation experimental group and a visual presentation experimental group. Ten participants (auditory presentation, 4) had experience attending Japanese universities as exchange students. Table 1 reported the N1 mean score, the self-report of Chinese and Japanese proficiency, the length of studying abroad, and Japanese usage frequency (referred to The Language History Questionnaire, LHQ-3, see Li et al., 2020) of participants in the case of different presentation modalities. The independent samples *t*-test for every indicator showed no significant difference between the two groups (*ps* > 0.05). Each condition excluded the influence of test differences on the experimental results as much as possible.

**TABLE 1:** Participants’ language proficiency, Japanese usage frequency, and comparisons of different presentation modalities.

			Auditory presentation	Visual presentation	*t*-test results		
					***t***	***p***	***cohen’d***
JLPT N1 score			152.83 (16.53)	148.72 (19.86)	0.68	0.504	0.23
Self-report proficiency scores	L	C	6.44 (0.51)	6.39 (0.50)	0.33	0.744	0.11
		J	5.28 (0.67)	5.11 (0.83)	0.66	0.512	0.22
	S	C	6.06 (0.64)	5.83 (0.62)	1.06	0.297	0.35
		J	4.61 (1.15)	4.50 (0.71)	0.35	0.728	0.12
	R	C	6.50 (0.51)	6.33 (0.49)	1.00	0.324	0.33
		J	5.67 (0.84)	5.89 (0.76)	0.83	0.411	0.28
	W	C	5.83 (0.79)	5.78 (0.65)	0.23	0.818	0.08
		J	4.78 (0.94)	5.06 (0.87)	0.92	0.365	0.31
Length of studying abroad (months)			2.06 (4.22)	2.83 (4.63)	0.53	0.602	0.18
Japanese usage frequency (hours/day)			4.11 (1.08)	4.39 (1.41)	0.66	0.511	0.22

The total score of JLPT N1 is 180. L, listening; S, speaking; R, reading; W, writing; C, Chinese; J, Japanese. 1, none ∼ 7, near native-like. Results are expressed as mean (*SD*).

After the experimenter’s detailed explanation of the study, all participants voluntarily signed the informed consent form, which clearly states that the experimental data will only be used for academic research and the personal information of the participants will never be disclosed to others. At the end of the experiment, all participants received a reward of 30 yuan.

### 3.2. Design

The present study employed a lexical decision task to compare our results with those of previous studies. Using linear mixed-effects models (LMMs), we aimed to examine the effects of semantic transparency and translational congruency on L2 Japanese collocational processing by advanced Chinese JFL learners. In our experimental design the presentation modality and semantic transparency, or the presentation modality and translational congruency, were fixed factors, respectively. To ensure that semantic transparency and translational congruency did not interfere with each other (Fang and Zhang, 2021), the semantic transparency experiment was conducted on collocations with congruent translation, while the translational congruency experiment was conducted on collocations with low semantic transparency.

### 3.3. Materials

Experimental materials were selected from three textbooks commonly used by Japanese majors in China (i.e., “ZongHe RiYu (Comprehensive Japanese), Peking University Press, 2007”; “XinBan ZhongRi JiaoLiu BiaoZhun RiBenYu (The New Edition of Standard Japanese for Sino-Japanese Communication), People’s Education Press and Mitsumura Tosho Publishing Co., Ltd., 2005”; “XinBian RiYu (Newly Compiled Japanese), Shanghai Foreign Language Education Press, 2009”). Collocations are multiword units that are more complex than words. Although the participants had attained JLPT N1 certificate, they were still unbalanced bilinguals, as indicated by their Japanese language proficiency self-assessment scores in Table 1. Considering the language ability of the participants and to ensure the psychological authenticity of the experimental material, collocations containing words from the JLPT N1 and words above the JLPT level were excluded.

According to the definition mentioned above and the classification of collocations, a total of 48 collocations in four conditions (12 collocations for each condition) were created (see Supplementary material). They were (A) collocations with high semantic transparency and congruent translation with L1, (B) collocations with medium semantic transparency and congruent translation with L1, (C) collocations with low semantic transparency and congruent translation with L1, and (D) collocations with low semantic transparency and incongruent translation with L1 (Table 2).

**TABLE 2:** Summary of characteristics of the test items.

Type	Log-transformed frequency of use	MI score	Mora	Familiarity	Semantic transparency	Example
A	3.14 (0.45)	9.88 (2.28)	6.92 (1.24)	6.52 (0.39)	5.78 (0.25)	自信を失う (jishin-wo-ushinau, lose confidence)
B	3.28 (0.44)	10.85 (2.17)	6.58 (1.08)	6.23 (0.42)	4.79 (0.88)	世話を焼く (sewa-wo-yaku, take care of someone)
C	2.88 (0.72)	10.81 (2.79)	6.25 (0.87)	6.14 (0.36)	3.78 (0.39)	耳を疑う (mimi-wo-utagau, be hard to believe)
D	2.81 (0.56)	10.07 (2.73)	6.25 (0.75)	6.14 (0.58)	3.49 (0.36)	油を売る (abura-wo-uru, loaf around)

The types of collocations were (A) collocations with high semantic transparency and congruent translation with L1, (B) collocations with medium semantic transparency and congruent translation with L1, (C) collocations with low semantic transparency and congruent translation with L1, and (D) collocations with low semantic transparency and incongruent translation with L1. Twelve collocations for each type of collocation were prepared as the test items. Frequency of use and MI score are based on Tsukuba Web Corpus. Chinese JFL learners rated familiarity. Japanese native speakers rated semantic transparency. Results are expressed as mean (*SD*).

The number of mora of collocation, the frequency of use by the Tsukuba Web Corpus (developed by the University of Tsukuba and the search engine is provided by Japan National Institute for Japanese Language and Linguistics), and the MI score (Mutual Information score, a measure of collocational strength) were calculated. Furthermore, one-way analysis of variance (ANOVA) was conducted for each characteristic. The results showed that no main effects were significant in any condition [number of mora: (*F*(3, 44) = 1.21, *p* = 0.316, η^2^ = 0.08); log-transformed frequency of use: (*F*(3, 44) = 1.88, *p* = 0.147, η^2^ = 0.11); MI score: (*F*(3, 44) = 0.48, *p* = 0.699, η^2^ = 0.03)]. We also asked 279 advanced Chinese learners of Japanese to rate the degree of familiarity with all collocations on a 7-point scale (1: not at all familiar ∼ 7: very familiar). One-way ANOVA was conducted on their mean ratings, and the main effect was not significant (*F*(3, 44) = 2.00, *p* = 0.128, η^2^ = 0.12).

Materials of semantic transparency and translational congruency are classified according to the results of research in the field of linguistics (Nattinger and DeCarrico, 1992; Miyoshi, 2007). Moreover, 23 Japanese L1 speakers were asked to rate the semantic transparency of the collocations in each condition on a 7-point scale (1: lowest semantic transparency ∼ 7: highest semantic transparency). One-way ANOVA was performed on the mean ratings of the four lists, and the main effect was significant (*F*(3, 44) = 46.48, *p* < 0.001, η^2^ = 0.76). Tukey’s HSD Test for multiple comparisons found that A > B > C≒D (A-B: *t*(44) = 4.58, *p* < 0.001, *cohen’s d* = 1.87; A-C: *t*(44) = 9.28, *p* < 0.001, *cohen’s d* = 3.79; A-D: *t*(44) = 10.58, *p* < 0.001, *cohen’s d* = 4.32; B-C: *t*(44) = 4.70, *p* < 0.001, *cohen’s d* = 1.92; B-D: *t*(44) = 6.01, *p* < 0.001, *cohen’s d* = 2.45; C-D: *t*(44) = 1.31, *p* = 0.565, *cohen’s d* = 0.53). Next, to reconfirm the reasonableness of the selection of congruent translational collocations (i.e., A, B, C) and incongruent translational collocations (i.e., D), we asked five advanced Chinese JFL learners to judge the materials selected by the authors based on the judgment of translational congruency, i.e., whether the Chinese and Japanese meanings were congruent. The result showed no objection to the selection of materials based on the judgment of translational congruency.

Finally, 3 lists, (A), (B), and (C), were used for the semantic transparency experiment, and 2 lists, (C) and (D), were used for the translational congruency experiment. In addition, 36 non-collocations (i.e., not exist in Japanese) were created for the lexical judgment task. The audio stimuli were recorded by a woman from the Greater Tokyo Area who had experience teaching Japanese, and the recordings were edited for auditory presentation.

### 3.4. Apparatus

The computer program used in the experiment was created using E-Prime 2.0 software. In the auditory presentation experiment, the auditory stimuli were presented through headphones. A personal computer and peripherals were used to present the computer program.

### 3.5. Procedure

**FIGURE 1:**
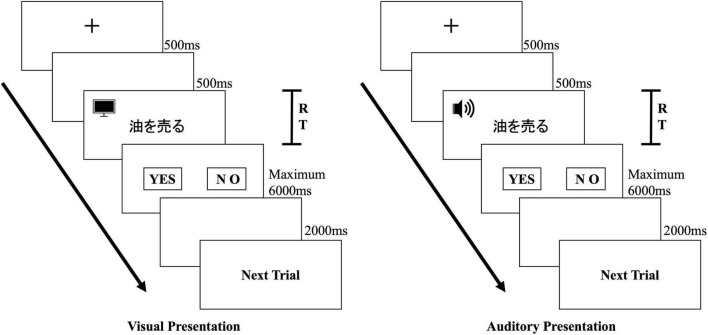
Flow of one experiment trial in the visual and auditory presentation conditions.

The experiments were conducted individually. Before starting the experiment, we conducted five practice sets to ensure that the participant understood the experimental procedure. Figure 1 shows the flow of the experiment for one trial.

In the visual presentation experiment, as a cue to present the collocation, the gaze point was presented on the computer screen for 500 ms. Then, after a blank space of 500 ms, the collocation was presented. Participants were instructed to judge whether the collocation presented on the computer screen was a correct Japanese collocation or not as quickly and accurately as possible, and to press the “Yes (*Z* key)” or “No (*M* key)” buttons, respectively. The collocation was presented for a maximum of 6000 ms. During that time, either the participant responded or, if no response was made and 6000 ms had elapsed, a blank screen was presented for 2000 ms, and then the next trial was started. The computer automatically measured the time from the start of the visual presentation until the participant pressed the key as the reaction time for the collocation.

In the auditory presentation experiment, as in the visual presentation experiment, after the gaze point was presented for 500 ms, a blank space of 500 ms was presented. Then, the participant listened to the collocation presented auditorily through headphones. Participants were instructed to judge whether the collocation they had heard was a correct Japanese collocation or not as quickly and accurately as possible, and to press the “Yes (*Z* key)” or “No (*M* key)” buttons, respectively. After the auditory presentation, if the participant had responded or if there was no response and 6000 ms had elapsed, a blank screen was presented for 2000 ms, and then the next trial was started. The computer automatically measured the time from the end of the auditory presentation until the participant pressed the key as the reaction time for the collocation.

In both the visual and auditory presentation experiments, the collocations were randomly presented by the computer program. After the completion of all trials, unknown collocations were checked by the participants. A written questionnaire about the participant’s language learning experiences was administered.

## 4. Results and discussion

### 4.1. Data trimming

Only correct responses to Yes trials were included in the analysis. The percentage of incorrect responses was 15.22%, and the percentage of more than 2.5*S Ds* beyond the mean and collocations the participants did not know was 3.82%. The incorrect responses and collocations the participants did not know were excluded from the analysis. To deal with the skewed data, reaction times were log-transformed. Tables 3, 4 show the results of the reaction times of correct responses to Yes trials and the accuracy rates in the semantic transparency and translational congruency experiments.

**TABLE 3:** Reaction times and accuracy rates in each condition of the semantic transparency experiment.

Variables		Reaction times (ms)	Accuracy rates (%)
Visual	Low semantic transparency	1341.17 (693.26)	85.19 (10.52)
	Medium semantic transparency	1312.84 (480.59)	82.48 (11.39)
	High semantic transparency	1203.09 (420.71)	93.52 (12.31)
Auditory	Low semantic transparency	2232.68 (720.41)	83.33 (9.48)
	Medium semantic transparency	2231.58 (707.50)	79.63 (14.64)
	High semantic transparency	2477.98 (829.72)	85.19 (17.04)

Results are expressed as mean (*SD*).

**TABLE 4:** Reaction times and accuracy rates in each condition of the translational congruency experiment.

Variables		Reaction times (ms)	Accuracy rates (%)
Visual	Incongruent translation	1256.99 (459.32)	79.63 (12.53)
	Congruent translation	1357.44 (628.08)	85.19 (10.52)
Auditory	Incongruent translation	1839.68 (482.59)	86.57 (10.36)
	Congruent translation	2180.70 (679.43)	83.33 (9.48)

Results are expressed as mean (*SD*).

Data analyses were conducted using the software R (version 4.2.1, R Core Team, 2022). We adopted linear-mixed effects modeling utilizing the lme4 (Bates et al., 2015) and lmerTest (Kuznetsova et al., 2017) packages. The emmeans package (Lenth, 2022) was used to examine interactions. The model with the lowest Akaike information criterion (AIC) was selected as the optimal model for model fitting. Wald *t*-distribution approximation was used to compute *p*-values for reaction times data. Wald *z*-distribution was used to compute *p*-values for the accuracy rates data. Since semantic transparency and translational congruency are related to the type of collocation, analyzing all stimulus items as random effects can improve the accuracy of the experimental results (Song and Fei, 2022).

### 4.2. Results and discussion of the semantic transparency experiment

**FIGURE 2:**
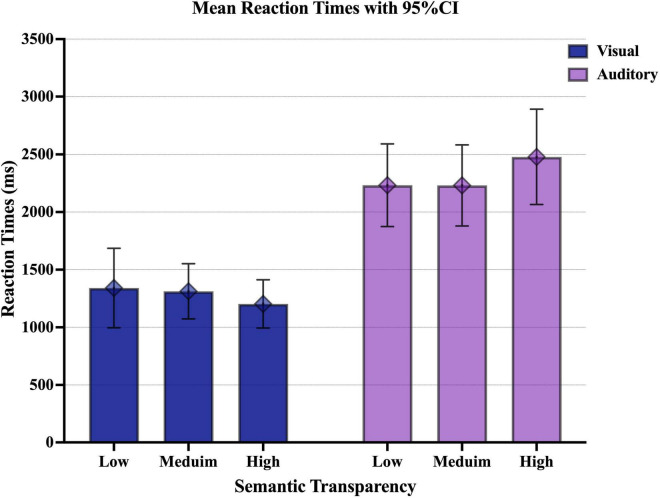
Graph of the mean reaction times upon visual or auditory presentation of Japanese collocations with different degrees of semantic transparency. The error bar shows the 95% confidence interval (CI).

Based on the AIC, semantic transparency, presentation modality, and the interaction between semantic transparency and presentation modality were selected as fixed effects and experimental participants and items were selected as random effects in the model. The analysis of results of the semantic transparency experiment are reported in Figure 2 and Table 5.

**TABLE 5:** Results of LME model analysis of reaction times in the semantic transparency experiment.

Variables	Estimate	SE	df	*t*-value	Pr (>|*t*|)
Intercept	3.07	0.03	56.04	113.56***	<0.001
modalityAud	0.32	0.04	50.26	8.88***	<0.001
conditionB	0.03	0.02	31.28	1.23	0.228
conditionC	0.03	0.02	31.01	1.45	0.156
conditionB: modalityAud	−0.08	0.03	32.68	−2.92**	0.006
conditionC: modalityAud	−0.08	0.03	31.64	−3.20**	0.003

**p < 0.01, ***p < 0.001. Participants = 36. Items = 36. Total observation = 1037. SE, standard error; df, degree of freedom. The optimal model is *lmer*(logrt∼modality × condition + (1|participant) + (modality|item), data = datAC).

In the semantic transparency experiment, the main effect of presentation modality was significant. The visual presentation condition was associated with shorter reaction times than the auditory presentation condition (*t*(50.26) = 8.88, *p* < 0.001). The main effect of semantic transparency was not significant (*p* > 0.100). Conversely, since the interaction between presentation modality and semantic transparency was significant, simple main effects were tested. Results indicated that in the auditory presentation condition, the reaction time for collocations with high semantic transparency tended to be longer than that for collocations with medium semantic transparency (*t*(33.0) = 1.96, *p* = 0.058) and was significantly longer than that for collocations with low semantic transparency (*t*(32.4) = 2.04, *p* = 0.050). There was no significant difference in reaction time between medium and low transparency collocations (*t*(33.0) = 0.29, *p* = 0.946). Meanwhile, it was shown that the effect of semantic transparency was not significant in the visual presentation condition [high/medium semantic transparency: (*t*(32.5) = 1.23, *p* = 0.228); high/low semantic transparency: (*t*(32.2) = 1.45, *p* = 0.156); medium/low semantic transparency: (*t*(33.5) = 0.22, *p* = 0.831)]. In addition, under all conditions, the reaction time upon visual presentation was significantly shorter than the reaction time upon auditory presentation [high semantic transparency: (*t*(50.6) = 8.88, *p* < 0.001); medium semantic transparency: (*t*(52.2) = 6.67, *p* < 0.001); low semantic transparency: (*t*(51.1) = 6.52, *p* < 0.001)].

**FIGURE 3:**
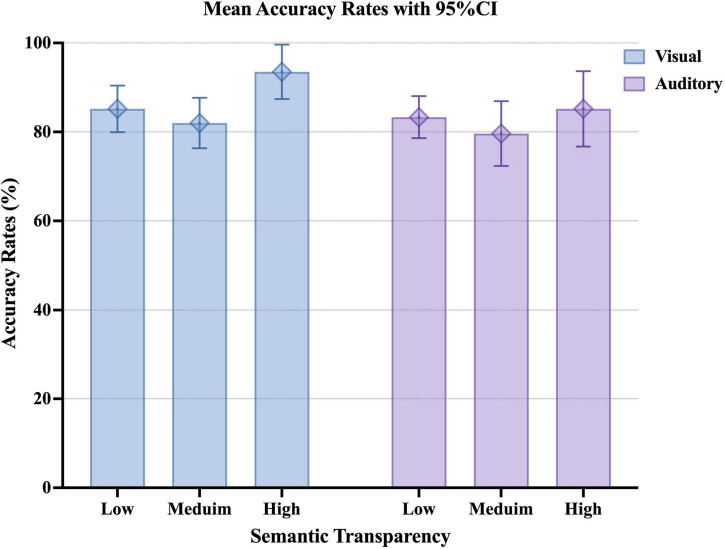
Graph of the mean accuracy rates upon visual or auditory presentation of Japanese collocations with different degrees of semantic transparency. The error bar shows the 95% CI.

We also analyzed the accuracy rates using the *glmer* function. The results showed that the main effect of presentation modality was significant, and the visual presentation condition was associated with higher accuracy rates than the auditory presentation condition (*z* = 2.41, *p* = 0.016) (Figure 3 and Table 6). The main effect of semantic transparency was significant. The accuracy rate was higher for collocations with high semantic transparency than for collocations with medium semantic transparency (*z* = 2.21, *p* = 0.027). Since the interaction between presentation modality and semantic transparency tended to be significant, simple main effects were tested. Results indicated that in the visual presentation condition, the accuracy rate tended to be higher for collocations with high semantic transparency than for those with medium semantic transparency (*z* = 2.22, *p* = 0.068). In addition, among collocations with high semantic transparency, the accuracy rate was significantly higher in the visual presentation condition than in the auditory presentation condition (*z* = 2.41, *p* = 0.016).

**TABLE 6:** Results of GLME model analysis of accuracy rates in the semantic transparency experiment.

Variables	Estimate	SE	*z*-value	Pr (>|*z*|)
Intercept	3.11	0.44	7.10***	<0.001
modalityAud	−1.00	0.41	−2.41*	0.016
conditionB	−1.17	0.53	−2.21*	0.027
conditionC	−0.83	0.54	−1.54	0.123
conditionB: modalityAud	0.80	0.43	1.84^†^	0.065
conditionC: modalityAud	0.85	0.45	1.89^†^	0.058

^†^*p* < 0.10, *p < 0.05, ***p < 0.001. Participants = 36. Items = 36. Total observation = 1296. SE, standard error. The optimal model is *glmer* (acc∼modality × condition + (1|participant) + (1|item), family = binomial, data = datAC).

These results indicate that presentation modality and semantic transparency interact in Japanese collocational processing. Based on these results, we will discuss the first research question of this study. The effect of semantic transparency in the auditory and visual presentation conditions differed, and it can be inferred that collocational processing differs depending on presentation modality. In the auditory presentation condition, the reaction time for collocations with high semantic transparency was longer than that for collocations with medium or low semantic transparency. This result is consistent with the results of studies of phonographic languages using the visual presentation condition (Brooks and Cid de Garcia, 2015; Chen et al., 2020).

In contrast, the effect of semantic transparency was less pronounced in the visual presentation condition. In the case of visual presentation, the presence of ideographic characters (Chinese characters) may have led to the superiority of decompositional processing, regardless of the degree of semantic transparency. In the auditory presentation condition, collocations with high semantic transparency were associated with the longest reaction times. This result may have been obtained because collocations with low semantic transparency are more likely to be processed as a whole than collocations with high semantic transparency.

The results about accuracy rates also showed that collocational processing differed depending on presentation modality. In the visual presentation condition, the accuracy rate tended to be higher for collocations with high semantic transparency than for those with medium semantic transparency. Meanwhile, the accuracy rate in the visual presentation condition was higher than that in the auditory presentation condition for collocations with high semantic transparency. These results reconfirmed the strong effect of L1. When collocations with high semantic transparency were visually presented to Chinese JFL learners, the learners could maximize the use of their knowledge of Chinese characters (e.g., Fei and Song, 2021).

Based on the above discussion, it can be said that when applying previous research results (e.g., Farrokh, 2012) to vocabulary teaching in order to promote the acquisition of collocations, if the students are Chinese JFL learners, the influences of presentation modality and L1 knowledge should also be taken into account.

### 4.3. Results and discussion of the translational congruency experiment

**FIGURE 4:**
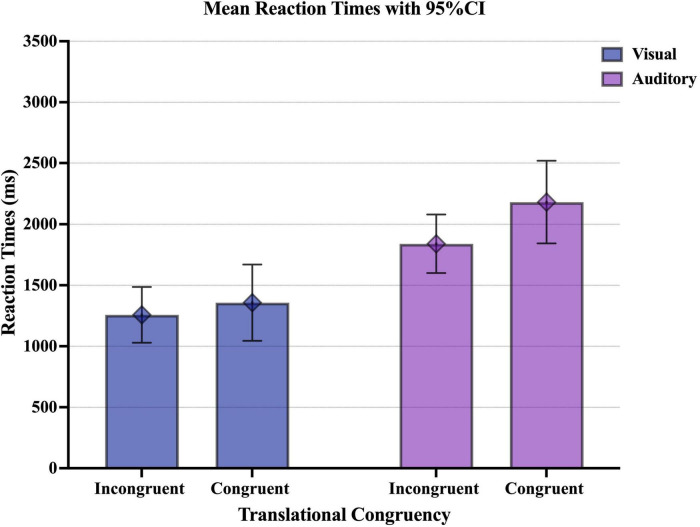
Graph of the mean reaction times upon visual or auditory presentation of Japanese collocations with low semantic transparency and different translational congruency. The error bar shows the 95% CI.

The results of the translational congruency experiment were analyzed (Figure 4 and Table 7). Based on the AIC, the model with translational congruency, presentation modality, and the interaction between translational congruency and presentation modality as fixed effects and experimental participants and items as random effects was determined to be the optimal model.

**TABLE 7:** Results of LME model analysis of reaction times in the translational congruency experiment.

Variables	Estimate	SE	df	*t*-value	Pr (>|*t*|)
Intercept	3.11	0.03	54.28	118.62***	<0.001
modalityAud	0.22	0.03	39.33	6.89***	<0.001
conditionD	−0.02	0.02	30.21	−0.89	0.381
conditionD: modalityAud	−0.05	0.02	629.62	−2.99**	0.003

**p < 0.01, ***p < 0.001. Participants = 36. Items = 24. Total observation = 687. SE, standard error; df, degree of freedom. The optimal model is *lmer*(logrt∼modality × condition + (1|participant) + (1|item), data = datCD).

The main effect of presentation modality was significant, and the reaction time was significantly longer in the auditory presentation condition than in the visual presentation condition (*t*(39.33) = 6.89, *p* < 0.001). The main effect of translational congruency was not significant, and there was no significant difference in reaction time between the congruent translational collocations and the incongruent translational collocations (*t*(39.33) = 0.89, *p* = 0.381). The interaction between translational congruency and presentation modality was significant. More specifically, in the auditory presentation condition, congruent translational collocations were associated with longer reaction times than incongruent collocations (*t*(29.4) = 2.83, *p* = 0.008). However, there was no significant difference in reaction time between congruent translational collocations and incongruent collocations in the visual presentation condition (*t*(29.9) = 0.89, *p* = 0.382). We also found that auditory presentation was associated with significantly longer reaction times than visual presentation, whether congruent translational collocations (*t*(39.4) = 6.89, *p* < 0.001) or incongruent translational collocations (*t*(39.2) = 5.33, *p* < 0.001) were presented.

**FIGURE 5:**
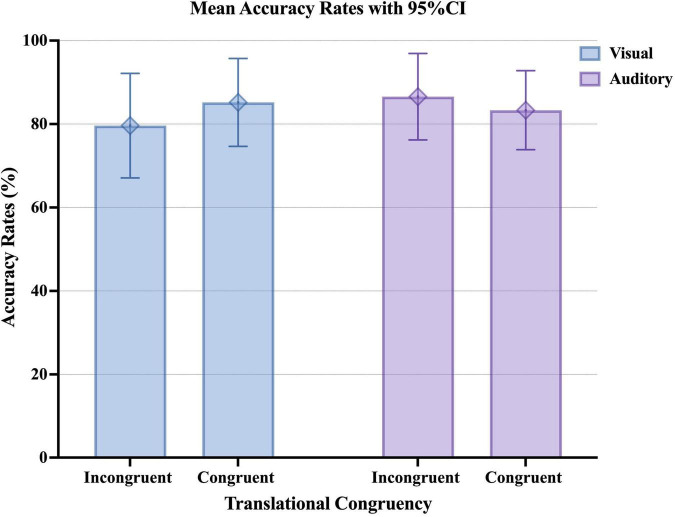
Graph of the mean accuracy rates upon visual or auditory presentation of Japanese collocations with low semantic transparency and different translational congruency. The error bar shows the 95% CI.

We also analyzed the accuracy rates using the *glmer* function. The results showed that the main effect of the presentation modality (*z* = 0.52, *p* = 0.604) and the main effect of translational congruency (*z* = 0.97, *p* = 0.331) were not significant (Figure 5 and Table 8). However, the interaction between presentation modality and translational congruency tended to be significant. As a result of simple main effects, the auditory presentation condition tended to be associated with higher accuracy rates than the visual presentation condition among incongruent translational collocations (*z* = 1.83, *p* = 0.068).

**TABLE 8:** Results of GLME model analysis of accuracy rates in the translational congruency experiment.

Variables	Estimate	SE	*z*-value	Pr (>|*z*|)
Intercept	2.27	0.42	5.40***	<0.001
modalityAud	−0.17	0.33	−0.52	0.604
conditionD	−0.54	0.55	−0.97	0.331
conditionD: modalityAud	0.77	0.40	1.92^†^	0.055

^†^*p* < 0.10, ***p < 0.001. Participants = 36. Items = 24. Total observation = 864. SE, standard error. The optimal model is *glmer* (acc∼modality × condition + (1|participant) + (1|item), family = binomial, data = datCD).

These results indicate that presentation modality and translational congruency interact in Japanese collocational processing. Based on these results, we will discuss the second research question of this study. In the visual presentation condition, the effect of translational congruency was weak, whereas, in the auditory presentation condition, the effect of translational congruency was strong. In the visual presentation condition, decompositional processing of syntactic analysis may have been dominant, regardless of the difference in translational congruency. This point differs from the results of studies on phonographic languages (Wolter and Gyllstad, 2011; Zhang, 2017). It can be seen that the influence of ideograms, i.e., Chinese characters, is stronger than that of phonetic characters. On the other hand, in the auditory presentation condition, incongruent translational collocations may have been predominantly processed as a whole.

In addition, the accuracy rate in the case of auditory presentation tended to be higher than that in the case of visual presentation only for incongruent translational collocations. Since the incongruent translational collocations all had low semantic transparency, it can be inferred that even collocations with low semantic transparency are affected differently by L1 and the presentation modality. These results further support the hypothesis that Chinese JFL learners process Japanese collocations in different ways in the case of different presentation modalities. Therefore, the presentation modality should be considered when discussing the Japanese collocational processing model in Chinese JFL learners.

## 5. General discussion

When Chinese JFL learners process Japanese *Kanji* words, their L1 knowledge of Chinese characters will be activated simultaneously and have a solid facilitative or inhibitory effect upon different presentation modalities (e.g., Matsumi et al., 2012; Hsieh et al., 2017, 2021; Fei et al., 2022). Therefore, it can be inferred that the Japanese collocational processing in Chinese learners may differ from that in learners of phonographic languages. In this study, we empirically investigated the effects of presentation modality, semantic transparency, and translational congruency on the processing of Japanese collocations by Chinese JFL learners. The experimental results revealed that these three factors are closely related to the processing of collocations, which complements the results of research on the processing of ideographic characters and provides a new empirical basis for the dual-route model.

**FIGURE 6:**
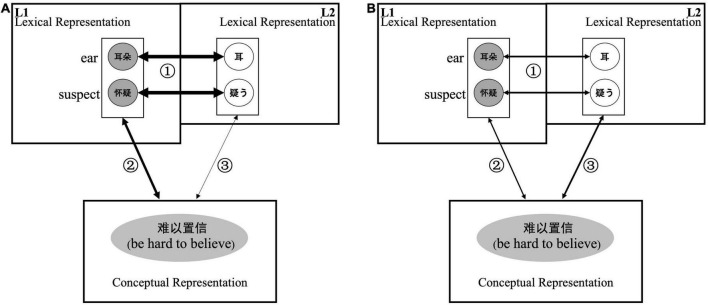
Processing of a congruent translational collocation in Chinese JFL learners. The thickness of the arrows indicates the strength of the links between representations. **(A)** Represents the processing of congruent translational collocation upon visual presentation. **(B)** Represents the processing of congruent translational collocation upon auditory presentation.

**FIGURE 7:**
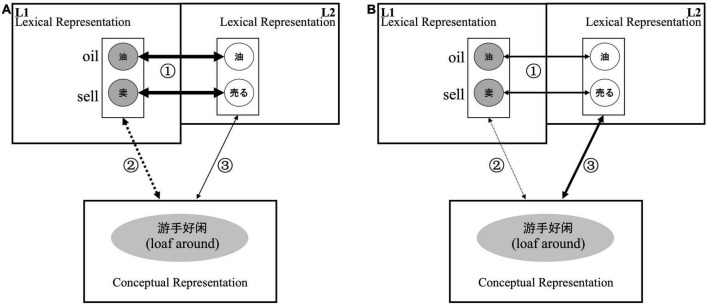
Processing of an incongruent translational collocation in Chinese JFL learners. The thickness of the arrows indicates the strength of the links between representations and the dotted arrows indicate that direct semantic access is impossible. **(A)** Represents the processing of incongruent translational collocation upon visual presentation. **(B)** Represents the processing of incongruent translational collocation upon auditory presentation.

Based on the results of this study and previous studies (i.e., the revised hierarchical model, Kroll and Stewart, 1994), we propose a Japanese collocational processing model for Chinese JFL learners in the case of different presentation modalities, as shown in Figure 6 (congruent translational collocations with different semantic transparencies) and Figure 7 (incongruent translational collocations with low semantic transparency). The thickness of the connecting arrows means the strength of the links between the representations, and the dotted arrows indicate that direct semantic access is impossible. We comprehensively examine the processing of collocations by Chinese learners of Japanese from the perspective of the influence of their knowledge of Chinese characters in the two languages and discuss the third research question of this study.

### 5.1. Effect of semantic transparency on collocational processing in the case of different presentation modalities

The effect of semantic transparency was not pronounced in the visual presentation condition. As mentioned above, all semantic transparency experiments in this study used congruent translational collocations. In other words, when the Chinese JFL learners received the visual input of Japanese collocations, the L1 semantics were rapidly activated, and the meaning of the collocation could be inferred from the L1 semantics. As shown in Figure 6, after visual presentation of the Japanese collocation, the L1 lexical representation was quickly activated, suggesting that semantic access *via* L1 was dominant (Figure 6A, ➀ → ➁).

In contrast, in the auditory presentation condition, the reaction time for collocations with high semantic transparency tended to be longer than the reaction time for collocations with medium semantic transparency and was significantly longer than the reaction time for collocations with low semantic transparency. The degree of activation of the L1 Chinese lexical representations may have been weaker in the auditory presentation condition than in the visual presentation condition. In addition, the degree of activation of L1 may differ depending on the level of semantic transparency. Based on these two reasons, it can be inferred that collocations with medium or low semantic transparency, in which the linkage between the constituent words is more robust than in collocations with high semantic transparency, have dominant semantic access to conceptual representations directly from Japanese phonological information (Figure 6B, line ➂).

The results of the present study support the existence of a dual-route model (e.g., Sosa and MacFarlane, 2002; Wolter and Gyllstad, 2011; Fang and Zhang, 2021), as claimed in previous studies. Furthermore, from the perspective of presentation modality, we were able to show a new way of interpreting the dual-route model. That is, in the visual presentation condition, the influence of ideographic Chinese characters was strong and changed the collocation processing pattern. As previously mentioned, studies on visual presentation of collocations in phonographic languages have shown that collocations with low semantic transparency dominate overall processing (Libben et al., 2003; Brooks and Cid de Garcia, 2015; Chen et al., 2020). However, according to the results of the present study focusing on Japanese collocational processing in Chinese JFL learners, there was no significant difference in reaction time upon visual presentation of collocations with different semantic transparency. Therefore, it can be further speculated that even collocations with low semantic transparency may be analytically processed by syntactic analysis upon visual presentation.

On the other hand, in the case of auditory presentation, the results were consistent with those of phonographic language studies. This indicates that the morphology of Chinese characters has a relatively weak effect on overall semantic processing upon auditory presentation of Japanese collocations. This differs from the results of research on the lexical processing of Japanese *Kanji* words (Fei et al., 2022). We can infer that the word combination changed the influence of Chinese characters on semantic processing to some extent. Therefore, collocations with low semantic transparency are more prone to full-listing processing at the semantic access stage.

### 5.2. Effect of translational congruency on collocational processing in the case of different presentation modalities

In the auditory presentation condition, congruent translational collocations were associated with longer reaction times than incongruent translational collocations. As discussed above, collocations with low semantic transparency have dominant semantic access from the Japanese lexical representation directly to the conceptual representation (Figure 6B, line ➂). Based on this result, it can be inferred that incongruent translational collocations have more dominant semantic access to the conceptual representation directly from the L2 lexical representation (Figures 7A, B, line ➂).

However, there was no significant difference in reaction times in the visual presentation condition, regardless of whether or not the translation between Chinese and Japanese was congruent. As mentioned above, conceptual representation access *via* L1 semantic representation was dominant in the visually presented condition, even for collocations with low semantic transparency. Since incongruent collocations cannot be accessed directly from the activated lexical representation in L1 (Figure 7A, ➀ → ➁), semantic access to the conceptual representation may be performed directly from the L2 Japanese lexical representation (Figure 7A, line ➂). Therefore, it can be inferred that the reaction time is lengthened by trying to access L1. Ultimately, there was no significant difference in reaction time for incongruent translational collocations compared with the reaction times for congruent translational collocations.

Similar to the semantic transparency results, it is clear that the influence of translational congruency differed depending on presentation modality. The results of the visual presentation condition were consistent with the results of the study of Fang and Zhang (2019) on Chinese learners of English. However, through the above analysis and discussion, the absence of a significant difference in reaction time was not due to the absence of the effect of L1 but due to the prolonged reaction times resulting from access to conceptual representations through L1 lexical representation.

Nevertheless, the results in the case of auditory presentation in the present study differ from the results reported in learners of phonographic languages in the visual presentation condition, the latter of which showed that the reaction times were shorter when processing congruent translational collocations (Yamashita and Jiang, 2010; Wolter and Gyllstad, 2011; Zhang, 2017). Chinese JFL learners spent longer reaction times when processing congruent translational collocations. Based on the experimental results of semantic transparency and translational congruency, in the auditory presentation condition, Chinese JFL learners were more likely to process Japanese collocations with low semantic transparency and incongruent translation as a whole (Figure 7B, line ➂).

### 5.3. Suggestions for teachers on Chinese JFL learners’ acquisition of Japanese collocations

Based on the experimental results and the discussion, we hereby put forward some suggestions on Japanese collocational acquisition by Chinese JFL learners.

Firstly, Japanese collocation teaching must pay attention to the influence of presentation modality. Within the scope of descriptive statistics, it can be seen that in the visual presentation modality situation, the collocations with low semantic transparency took longer reaction times, while in the auditory presentation modality situation, the collocations with high semantic transparency took longer reaction times. The influence of semantic transparency on auditory presentation modality is more substantial than that on visual presentation modality. Chinese JFL learners tend to rely on visual information in Japanese language acquisition and therefore need to pay attention to auditory information-obtaining exercises (e.g., Fei et al., 2022), the lack of which may result in slower development of listening comprehension. In this regard, we propose two suggestions. First, teachers must remind learners to do more targeted auditory information-capturing exercises. Visual information is compared with auditory information to strengthen the linkage in terms of orthography, phonology, and semantics. The second suggestion is to intensify collocational practices. Even for collocations with high semantic transparency, teachers still need to list several common collocations, such as “石を投げる (ishi-wo-nageru), throw stones,” “石を拾う (ishi-wo-hirou), pick up stones,” “石を積む (ishi-wo-tsumu), pile up stones,” “石を運ぶ (ishi-wo-hakobu), carry stones,” etc. By strengthening visual and auditory exercises, the processing of collocations with high semantic transparency is upgraded to holistic processing.

Secondly, Japanese collocation teaching must pay attention to the influence of L1. In both cases of visual and auditory presentation modalities, the reaction times for collocations with congruent translation were longer. This suggests that L1 is activated and has an impact on the processing of congruent translational collocations. Especially in the case of visual presentation modality, learners can quickly understand the meaning with the aid of L1, while ignoring the integrity of collocations. Moreover, the accuracy rates for collocations with incongruent translation in the case of visual presentation modality were the lowest. For instance, the meaning of “油を売る (abura-wo-uru), loaf around” has no relation to the meanings of “油 (abura), oil” and “売る (uru), sell.” Due to the strong visual influence of L1, Chinese learners are more likely to make mistakes in the quick lexical decision of collocations. In addition, some participants made wrong responses to the two collocations of “困難を開く” and “興味を上げる” as fillers, thinking they were correct Japanese collocations, even though they do not exist in Japanese. Based on these results, we infer that this may also be due to the negative transfer of their L1 because there are two collocations of “解开困局 (jiekai-kunju), untie the dilemma” and “提高兴趣 (tigao-xingqu), enhance interest” in Chinese. However, since Chinese learners cannot wholly exclude the influence of L1, teachers’ advice to “avoid the influence of L1 as much as possible” may backfire and confuse learners. Therefore, it is recommended that teachers point out the difference between L1 Chinese and L2 Japanese to help learners understand the similarities and differences of the two languages. By understanding the characteristics of Chinese-Japanese bilingual vocabulary, learners can further strengthen the linkage between the orthography, phonology, and semantics of L2 and upgrade the processing of collocations with congruent translation to the level of holistic processing.

The above suggestions for teachers aim to comprehensively improve learners’ L2 processing efficiency and understanding. They improve the efficiency of visual information processing and help learners with language use in reading, test taking, and other scenarios. Likewise, they improve the efficiency of auditory information processing and help learners to apply language knowledge in listening comprehension, communication, and other activities.

## 6. Conclusion

In this study, based on the findings of previous studies, we comprehensively examined the processing of Japanese collocations by Chinese JFL learners. The results revealed that the linguistic characteristics of collocations in Japanese and the relationship between the two languages strongly influence the processing of Japanese collocations. Moreover, the influence of these two factors is closely related to presentation modality, such as visual or auditory presentation.

Learning and memorizing as a whole is generally advocated for the acquisition of L2 collocations. However, according to the results of this study, in the process of acquisition of Japanese collocations by Chinese JFL learners, it seems necessary to find ways to treat the relevance of their knowledge of Chinese characters and Japanese *Kanji* words correctly. We await further empirical studies using eye-tracking devices to investigate these issues, mainly focusing on the relationship between the Chinese and Japanese languages for each constituent word.

## Data availability statement

The raw data supporting the conclusions of this article will be made available by the authors, without undue reservation.

## Ethics statement

Ethical review and approval was not required for the study on human participants in accordance with the local legislation and institutional requirements. The patients/participants provided their written informed consent to participate in this study.

## Author contributions

QS, XF, and NM contributed to the conception of the work. QS and XF collected the experimental data. QS conducted the analysis and wrote the first manuscript. All authors revised the manuscript and confirmed its final version.
