# Supplementary material for: The lexical processing of Japanese collocations by Chinese Japanese-as-a-Foreign-Language learners: An experimental study by manipulating the presentation modality, semantic transparency, and translational congruency
Source: Front Psychol. 2023 Apr 4;14:1142411. doi: 10.3389/fpsyg.2023.1142411 (PMC10111037; doi:10.3389/fpsyg.2023.1142411)
Supplement: Supplementary file 1 [file Data_Sheet_1.PDF]

Supplementary Material 1　Collocations for the Experiment

Type	Collocation	Meaning		
		Overall	Former Constituent Word	Latter Constituent Word※
A	罪を犯す	*commit a crime*	*crime*	*commit*
A	説明を省く	*omit explanation*	*explanation*	*omit*
A	許可を得る	*get permission*	*permission*	*get*
A	原因を探る	*find the cause*	*cause*	*find*
A	目を閉じる	*close eyes*	*eye*	*close*
A	席を譲る	*offer one's seat*	*seat*	*offer*
A	自信を失う	*lose confidence*	*confidence*	*lose*
A	温度を下げる	*lower the temperature*	*temperature*	*lower*
A	順番を待つ	*wait for one's turn*	*turn*	*wait for*
A	負担を減らす	*reduce the burden*	*burden*	*reduce*
A	生活を楽しむ	*enjoy life*	*life*	*enjoy*
A	涙を流す	*shed tears*	*tear*	*shed*
B	注目を浴びる	*attract attention*	*attention*	*be bathed in*
B	技術を磨く	*improve one's skills*	*skill*	*polish*
B	焦点を当てる	*focus on*	*focus*	*hit*
B	鍵をかける	*lock up*	*key*	*fasten*
B	ため息をつく	*heave a sigh*	*sigh*	*breathe*

Type	Collocation	Meaning		
		Overall	Former Constituent Word	Latter Constituent Word※
B	汗をかく	*sweat*	*sweat*	*scratch*
B	注意をはらう	*pay attention*	*attention*	*pay*
B	習慣をつける	*form a habit*	*habit*	*attach*
B	恨みを買う	*incur someone's enmity*	*enmity*	*buy*
B	音をたてる	*make a sound*	*sound*	*make stand*
B	世話を焼く	*take care of someone*	*care*	*burn*
B	理由をつける	*make up a reason*	*reason*	*attach*
C	顔が広い	*be widely known*	*face*	*wide*
C	気が進まない	*be reluctant to do*	*spirit*	*don't move forward*
C	耳が痛い	*be ashamed to hear something*	*ear*	*painful*
C	足を運ぶ	*show up*	*foot*	*carry*
C	筋が通る	*make sense*	*tendon*	*go through*
C	心を打つ	*touch a person's heart*	*heart*	*hit*
C	手が届く	*take good care of*	*hand*	*reach*
C	心に刻む	*keep in one's heart*	*heart*	*carve*
C	耳を疑う	*be hard to believe*	*ear*	*suspect*
C	心が小さい	*small-minded*	*heart*	*small*
C	棚に上げる	*shut one's eyes to*	*shelf*	*raise*
C	顔を出す	*make an appearance*	*face*	*put out*

Type	Collocation	Meaning		
		Overall	Former Constituent Word	Latter Constituent Word※
D	首になる	*be fired*	*neck*	*become*
D	頭に来る	*get angry*	*head*	*come*
D	肩を入れる	*take sides with*	*shoulder*	*insert*
D	心を配る	*give attention to*	*heart*	*deliver*
D	腕を磨く	*develop one's skills*	*arm*	*polish*
D	調子に乗る	*feel cocky*	*pitch*	*ride*
D	手も足も出ない	*at one's wit's end*	*hand and foot*	*don't come out*
D	油を売る	*loaf around*	*oil*	*sell*
D	足を洗う	*turn over a new leaf*	*foot*	*wash*
D	腹をたてる	*get angry*	*belly*	*make stand*
D	頭が下がる	*admire greatly*	*head*	*fall*
D	空気を読む	*read the situation*	*air*	*read*

※ *"つける" and "かける" are polysemous words, and the basic meanings are listed here.*

It should be noted that the judgment of translational congruency is based on Chinese semantics. Therefore, the judgment of some collocations cannot match the English meaning listed above. For example, "棚に上げる" is an idiom that is consistent with Chinese (束之高阁, shu-zhi-gao-ge), so even though it is difficult to infer or translate the overall meaning of each constituent word, it is classified as congruent translational collocation due to the relevance of Chinese-Japanese bilingualism.

Supplementary Material 2　Fillers for the Experiment

Filler		
約束を買う	窓を落とす	散歩を探る
タバコを食べる	薬を食べる	時間を触る
新聞を消す	タクシーを打つ	口が育つ
ゴミを考える	頭が嬉しい	咳をひく
音楽を読む	怪我を指す	料理を立てる
映画を歌う	友人を交際する	花火をいう
残業が笑う	お風呂を行う	見学を見る
お金を話す	効果を見つける	困難を開く
言語を流す	責任を鳴らす	テストが細い
興味を上げる	ストレスを下げる	腹を持つ
英語を了解する	語感を飛ばす	視線を転移する
写真をかく	旅行を作る	文句が大きい
